# Modified Omega-k Algorithm for High-Speed Platform Highly-Squint Staggered SAR Based on Azimuth Non-Uniform Interpolation

**DOI:** 10.3390/s150203750

**Published:** 2015-02-05

**Authors:** Hong-Cheng Zeng, Jie Chen, Wei Liu, Wei Yang

**Affiliations:** 1 School of Electronic and Information Engineering, Beihang University, Beijing 100191, China; E-Mails: zenghongcheng@buaa.edu.cn (H.-C.Z.); chenjie@buaa.edu.cn (J.C.); 2 Electronic and Electronic Engineering Department, University of Sheffield, Sheffield S1-3JD, UK; E-Mail: w.liu@sheffield.ac.uk

**Keywords:** staggered SAR, continuous PRI variation, azimuth non-uniform sampling (ANS), modified Omega-k

## Abstract

In this work, the staggered SAR technique is employed for high-speed platform highly-squint SAR by varying the pulse repetition interval (PRI) as a linear function of range-walk. To focus the staggered SAR data more efficiently, a low-complexity modified Omega-k algorithm is proposed based on a novel method for optimal azimuth non-uniform interpolation, avoiding zero padding in range direction for recovering range cell migration (RCM) and saving in both data storage and computational load. An approximate model on continuous PRI variation with respect to sliding receive-window is employed in the proposed algorithm, leaving a residual phase error only due to the effect of a time-varying Doppler phase caused by staggered SAR. Then, azimuth non-uniform interpolation (ANI) at baseband is carried out to compensate the azimuth non-uniform sampling (ANS) effect resulting from continuous PRI variation, which is further followed by the modified Omega-k algorithm. The proposed algorithm has a significantly lower computational complexity, but with an equally effective imaging performance, as shown in our simulation results.

## 1. Introduction

Synthetic Aperture Radar (SAR) has become an indispensable part of current Earth observation systems [1–3]. Recently, highly-squint SAR onboard high-speed platforms (such as unmanned aerial vehicles or missiles) has developed very quickly and been employed widely for target detection, natural disaster monitoring, and surveillance, *etc.* [3–8]. However, the spatial-variant range cell migration (RCM) problem in highly-squint high-speed SAR data is much more challenging than in the traditional side-looking SAR mode, leading to not only extremely large data size and computational load [9–11], but also difficulty in acquiring the radar echo signals.

To mitigate the RCM problem, we can have a large pulse repetition interval (PRI) and change the opening time of the receive-window to remove the range-walk term in RCM [12,13]. However, this is not applicable to high-speed platform highly-squint SAR due to the lower PRI caused by the larger Doppler bandwidth. Another method is to continuously vary the PRI, which is referred to as staggered SAR [14,15]. This concept was first introduced for imaging with a wide seamless coverage by smoothly shifting the blind ranges across the swath with continuous PRI variation [16,17]. It has also been applied to highly-squint high-speed platform SAR to mitigate the large range-walk effect and solve the range blinding problem [18].

In this work, we focus on the high-speed platform highly-squint staggered SAR and vary the PRI as a function of range-walk to effectively receive the radar echo signals. However, this results to two problems: azimuth non-uniform sampling (ANS) and Doppler phase history changing (DPHC). The DPHC problem can be overcome by recovering the RCM back into the original form through zero padding in the range direction [19]. Then, the ANS effect can be compensated by azimuth non-uniform interpolation (ANI) at baseband, as the azimuth spectrum is centered at non-zero frequency in squint mode. At last, to focus signals accurately, we can employ the classic Omega-k algorithm [20,21]. However, the traditional algorithm is very time-consuming and requires large storage due to zero padding [22].

Therefore, in this paper a modified Omega-k algorithm based on ANI is proposed. An approximate model on continuous PRI variation with respect to sliding receive-window is employed, with a residual phase error only due to effect of a time-varying Doppler phase caused by different sampling positions of the platform. And the effect of ANS is removed by optimal ANI processing at baseband. Then, considering the removal of range-walk resulting from the continuous PRI variation, modified bulk compression and Stolt interpolation is derived. Consequently, the proposed algorithm can be implemented successfully without any data extension through zero padding.

This paper is organized as follows. Details of the high-speed platform highly-squint staggered SAR are provided in Section 2, while we focus on the proposed algorithm in Section 3, where an overview of the traditional algorithm is first presented in Section 3.1, followed by the proposed modified Omega-k algorithm in Section 3.2. An analysis of the computational complexity of the proposed algorithm is given in Section 4.1 and simulation results are provided in Section 4.2. Finally, conclusions are drawn in Section 5.

## 2. High-Speed Platform Highly-Squint Staggered SAR

**Figure 1.**
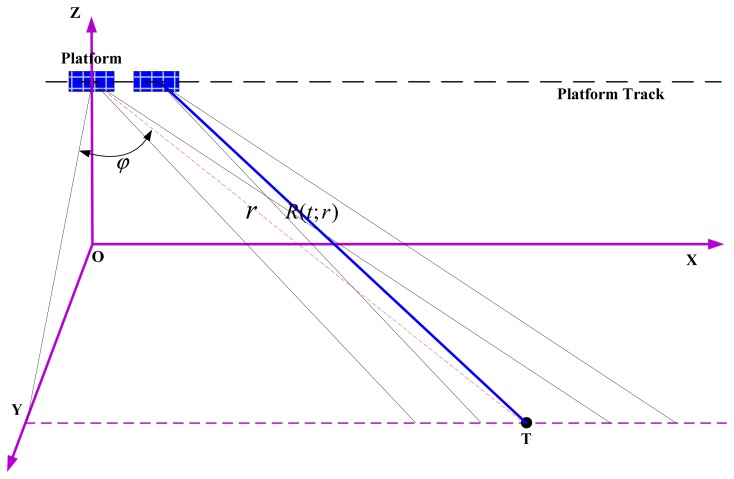
Imaging geometry of highly-squint high-speed platform SAR.

Figure 1 shows the imaging geometry of highly-squint high-speed platform SAR. Point O is the nadir at the azimuth time zero. The platform travels along the azimuth direction, parallel to the x-axis, and the y-axis points along the range direction. Furthermore, φ is the squint angle, *R*(*t;r*) denotes the instantaneous slant range distance from the antenna phase center (APC) to a certain target T, and *r* is the range.

The instantaneous slant range *R*(*t*) for target T can be expressed as [21]:

(1) $$\begin{array}{ll} R(t) & =\sqrt{r^{2}+v^{2}t^{2}-2\text{rvt}\sin\varphi }\\ & =\underset{\text{range}‐\text{walk}}{\underset{︸}{r-vt\sin\varphi }}+\underset{\text{range}‐\text{curve}}{\underset{︸}{\frac{v^{2}t^{2}\cos^{2}\varphi }{2r}+\cdots }} \end{array}$$

where *v* is the velocity of the platform, and *t* is the azimuth time. The range-walk term in Equation (1) is independent of slant range, while the following term represents the range-curve, which is much smaller than the range-walk [18].

**Figure 2.**
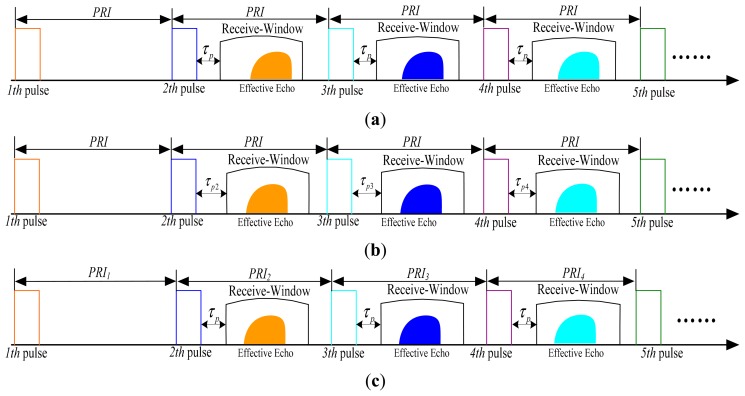
Different SAR working modes. (**a**) Constant PRI and fixed receive-window; (**b**) Constant PRI and sliding receive-window; (**c**) Variable PRI and fixed receive-window.

In a traditional SAR system, it transmits and receives linear frequency-modulated (LFM) signals with a constant PRI and a fixed receive-window opening delay time, as shown in Figure 2a. However, the RCM will increase significantly at highly-squint angles, and its length can be much larger and even exceed the size of receive window. As the range-walk is not only the prominent part of RCM but also independent of the target, we can use two methods to alleviate the RCM. One is to slide the receive-window opening time to remove the range-walk, as shown in Figure 2b, and a larger PRI is needed [12,13]; the other one is using continuous PRI variation with staggered SAR to remove the range-walk, as Figure 2c shows.

**Figure 3.**
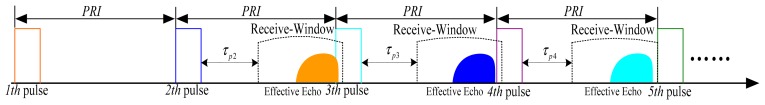
An example when the sliding receive-window method is not working.

For high-speed platform highly-squint SAR, the length of continuous reception-time limits its echo data acquisition, since RCM increases with squint angle. Furthermore, with the lower PRI caused by highly-squint angle and high-speed of the platform, there is even no enough space to shift the receive-window, rendering the sliding receive-window method invalid as shown in Figure 3. However, this is not the case for staggered SAR. For staggered SAR, even though any given transmitted pulse is unable to be received until several pulse intervals have elapsed, it is no longer an issue for highly-squint high-speed platform SAR. Therefore, the staggered SAR is used for solving the problem of echo acquisition limitation.

According to Equation (1), the function of range-walk with time *t* is:

(2) $$R_{w}(t)=-vt\sin\varphi$$

**Figure 4.**
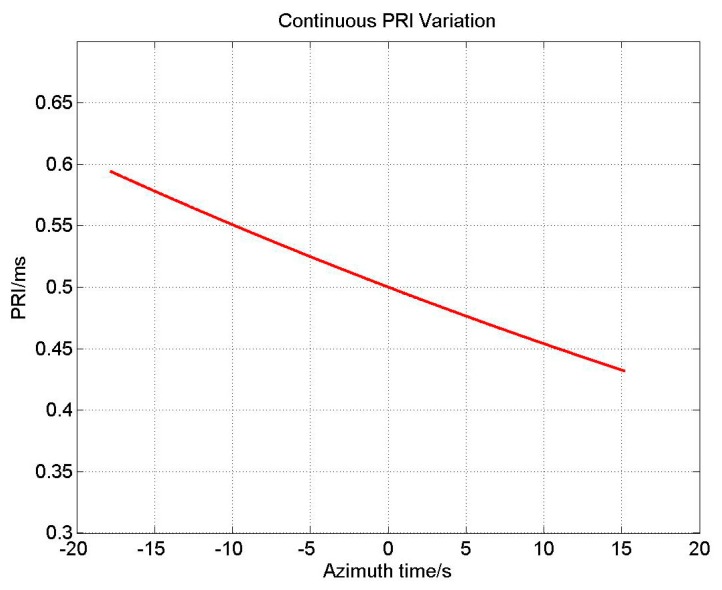
PRI variation with respect to the azimuth time.

As range-walk is independent of slant range, in the calculation of staggered SAR, we can remove the range-walk term first and the relationship between the (*i +* 1)th and the *i*th transmitted pulse intervals *PRI_i + 1_* and *PRI_i_* can be revised as [18]:

(3) $$PRI_{i+1}=\left(1-\frac{2v\sin\varphi }{c}\right)PRI_{i}$$

where *c* is the speed of light. Figure 4 shows PRI variation with respect to the azimuth time using parameters listed in Table 1 (in Section 4). With continuous variation of PRI, the azimuth sample positions keep changing, causing ANS effect on azimuth data.

## 3. Processing Algorithm for High-Speed Platform Highly-Squint Staggered SAR

In this section, the traditional algorithm for high-speed platform highly-squint staggered SAR is introduced first, and then, based on the similarity of staggered SAR and sliding receive-window SAR, a modified Omega-k algorithm employing ANI is proposed.

In order to facilitate the discussion and mathematical formulation, the following assumptions are made:

- The sensor trajectory is linear;
- The platform velocity is constant;
- The “stop-go” approximation is adopted.

### 3.1. Traditional Algorithm for High-Speed Platform Highly-Squint Staggered SAR

With the analysis of Section 2, the instantaneous slant range for target T in staggered SAR can be rewritten as:

(4) $$\begin{array}{cc} R(t_{i})=\sqrt{r^{2}+v^{2}t_{i}^{2}-2rvt_{i}\sin\varphi }, & t_{i}=\overset{n=i-1}{\underset{n=1}{\text{∑}}}PRI_{n},i\geq 2;\;t_{1}=0 \end{array}$$

where *t_i_* denotes the *i*th transmitted pulse time. The demodulated baseband SAR signal, *S_0_*(τ, *t_i_; r*), received from the single point target T can be modeled as [13]:

(5) $$S_{0}(\tau ,t_{i})=\omega \left(\tau -\frac{2R(t_{i})}{c}\right)\exp\left\{-j\pi K_{r}{\left[\tau -\frac{2R(t_{i})}{c}\right]}^{2}\right\}\exp\left\{-j\frac{4\pi R(t_{i})}{\lambda }\right\}$$

where ω(·), τ, *K_r_* and λ denote signal envelope, range time, range chirp FM rate and signal wavelength, respectively. To simplify the derivation, the backscatter coefficient and amplitude factors have been ignored.

**Figure 5.**
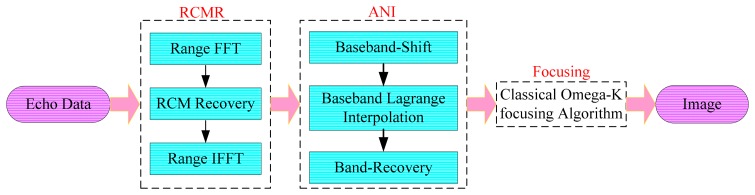
Processing details of the traditional imaging algorithm for staggered SAR.

To tackle the inherent problems of DPHC and ANS in staggered SAR, the imaging algorithm normally includes three stages: range cell migration recovery (RCMR), interpolation and focusing. Details for each stage are shown in Figure 5.

At the RCMR stage, the recovery of RCM is performed by phase compensation with filter Equation (6), in range-frequency and azimuth-time domain [19]:

(6) $$H_{\text{rcmr}}(f_{\tau },t_{i},t)=\exp\left\{j2\pi f_{\tau }\Delta t_{i}\right\}\exp\left\{-j4\pi \frac{R(t_{i})-R(t)}{c}f_{\tau }\right\}$$

(7) $$\Delta t_{i}=t_{i}-t=\left(\frac{2R(t_{i})}{c}\right)\mathrm{mod}\left(PRI_{i}\right)-\left(\frac{2R(t)}{c}\right)\mathrm{mod}\left(PRI_{0}\right)$$

where *f*_τ_ is the range frequency, *PRI_0_* is the mean PRI of the sequence of varied PRI, *t* is the azimuth uniform sampling time and mod(·) the complementation function.

At ANI stage, traditional Lagrange interpolation is performed instead of non-uniform FFT, as the non-uniform FFT processing is more complicated and the processing result is highly dependent on parameter selection [23]. Since the target spectrum is centered at non-zero frequency in squint mode, the azimuth signal should be moved to baseband before Lagrange interpolation. After interpolation, the data is resampled to a uniform grid, so that the target spectrum is recovered back to its original center frequency *fd_0_*, and $fd_{0}=-\;\overset{2v\cos\varphi }{\;}/\underset{\lambda }{\;}$. More details of this stage will be provided in Section 3.2.

In the focusing stage, the classic Omega-k focusing algorithm is implemented [20,24]. Bulk compression is applied by the filter *H_Bulk_*(*f_τ_*, *f*):

(8) $$H_{\text{Bulk}}(f_{\tau },f)=\exp\left\{-j\pi \frac{f_{\tau }^{2}}{K_{r}}\right\}\exp\left\{j\frac{4\pi R_{\text{ref}}}{c}\left[\sin\varphi \cdot \sqrt{{\left(f_{\tau }+f_{0}\right)}^{2}-{\left(\frac{cf}{2v}\right)}^{2}}+\cos\varphi \cdot \frac{cf}{2v}\right]\right\}$$

where *f_0_*, *f* and *R_ref_* denote the signal carrier frequency, azimuth frequency and reference range, respectively. And the new range frequency of Stolt interpolation is:

(9) $$f_{\tau }^{\prime }+f_{0}=\sin\varphi \cdot \sqrt{{\left(f_{\tau }+f_{0}\right)}^{2}-{\left(\frac{cf}{2v}\right)}^{2}}+\cos\varphi \cdot \frac{cf}{2v}$$

Then, a two-dimensional IFFT is performed to transform the signal back to the time domain. Finally, the staggered SAR echoes are focused accurately.

### 3.2. A Modified Omega-k Algorithm Based on ANI

**Figure 6.**
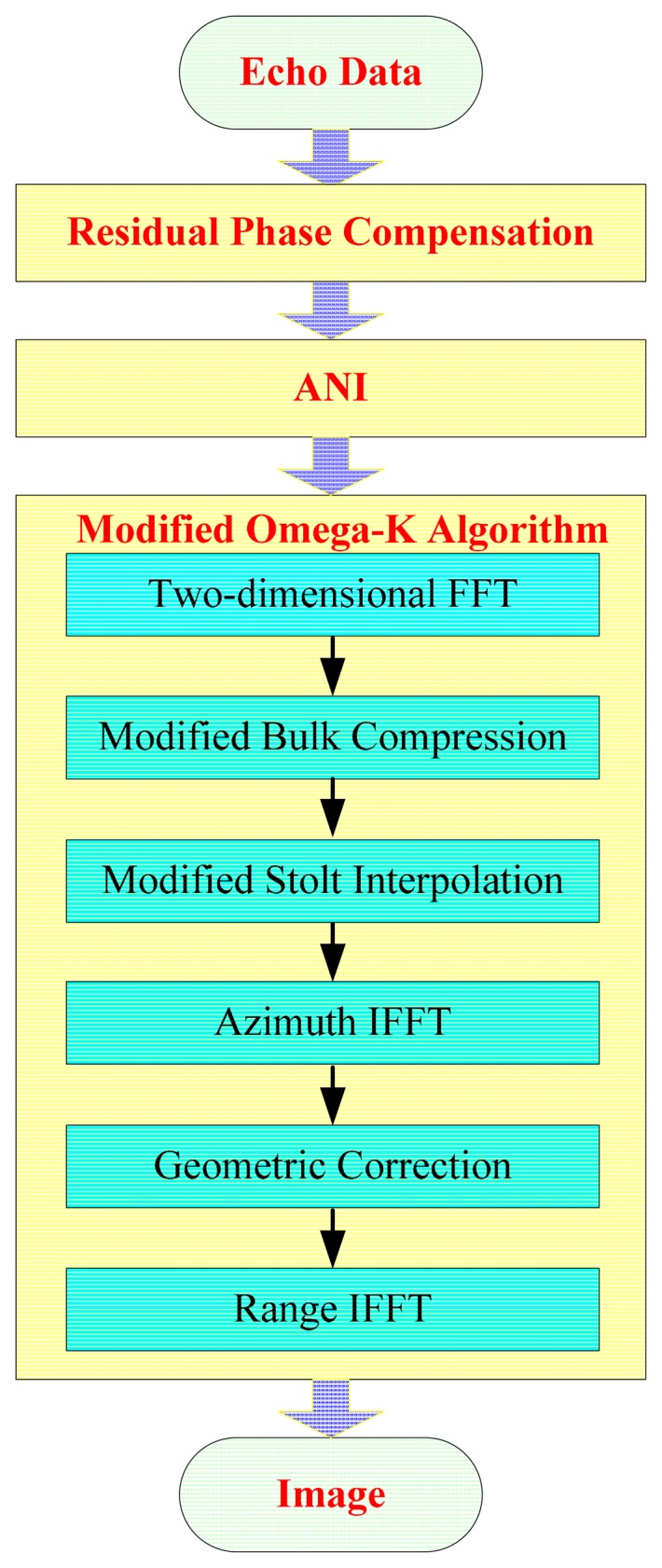
Block diagram of the modified Omega-k algorithm based on ANI.

Although the traditional algorithm is accurate enough for staggered SAR image formation, it is very time-consuming and requires large storage for processing. In this part, an approximate model on continuous PRI variation with respect to sliding receive-window is employed in the proposed algorithm, leaving a residual phase error only due to the effect of a time-varying Doppler phase caused by staggered SAR. As its block diagram is shown in Figure 6, where different from the traditional algorithm, the processing for range-walk recovery is no longer needed and the inherent signal characteristics in staggered SAR are preserved.

We assume that the post-ANI processing data acquired by the SAR mode with a sliding receive-window aiming for range-walk removal and the PRI is constant, as indicated in Figure 2b. However, different from traditional SAR, the platform sampling position of staggered SAR is accordingly changing due to varying PRI. For this approximation model, it only leaves a residual phase error with the different range history caused by staggered SAR. This residual phase error should be compensated before the ANI by the following filter:

(10) $$H_{r}(t_{i},t)=\exp\left\{-j4\pi \frac{R(t_{i})-R(t)}{\lambda }\right\}$$

Then, ANI processing is performed to resample the azimuth signal into a uniform grid, and its processing steps are identical to the traditional method, as shown in Figure 5. In the high-speed platform highly-squint staggered SAR, the azimuth spectrum is centered far away from the zero-frequency. As a result, the normal uniform interpolation inevitably leads to resolution degradation and leak of echo energy. Therefore, baseband non-uniform interpolation is needed for a quality imaging result. The first step of the ANI stage is moving the azimuth spectrum center to zero-frequency for baseband Lagrange interpolation through multiplying a linear phase term *H_base_*(*t_i_*) [25]:

(11) $$H_{\text{base}}\left(t_{i}\right)=\exp\left\{-j2\pi fd_{0}t_{i}\right\}$$

where *fd_0_* is Doppler centroid. Then the baseband Lagrange interpolation is performed to resample the data to a uniform grid. After that, the target spectrum should be recovered back to its original center frequency *fd_0_*, by multiplying *H_recovery_*(*t*) [25]:

(12) $$H_{\text{recovery}}\left(t\right)=\exp\left\{j2\pi fd_{0}t\right\}$$

After the ANI processing, the single point target signal can be expressed as [12]:

(13) $$S_{1}(\tau ,t)=\omega \left(\tau -\frac{2R(t)}{c}-\Delta T(t)\right)\cdot \exp\left\{-j\pi K_{r}{\left[\tau -\frac{2R(t)}{c}-\Delta T(t)\right]}^{2}\right\}\cdot \exp\left\{-j\frac{4\pi R(t)}{\lambda }\right\}$$

(14) $$\Delta T(t)=\frac{2vt\sin\varphi }{c}$$

where *v* is a constant for the platform. Applying FFT with respect to *τ*, the signal *S_1_*(τ, *t*) is transformed into the range frequency domain, yielding:

(15) $$\begin{array}{ll} S_{2}(f_{\tau },t) & =\int S_{1}(\tau ,t)\exp\left\{-j2\pi f_{\tau }\tau \right\}d\tau \\ & =\omega \;\left[-\frac{f_{\tau }}{K_{r}}\right]\cdot \exp\left\{j\pi \frac{f_{\tau }^{2}}{K_{r}}\right\}\cdot \exp\left\{-j\frac{4\pi \left(f_{0}+f_{\tau }\right)R(t)}{c}\right\}\\ & \cdot \exp\left\{-j\frac{4\pi vt\sin\varphi }{c}f_{\tau }\right\} \end{array}$$

Then, to obtain two-dimensional (2D) spectrum of the signal, the azimuth FFT is applied to Equation (15):

(16) $$\begin{array}{ll} S_{3}(f_{\tau },f) & =\int S_{2}(f_{\tau },t)\cdot \exp\left\{-j2\pi ft\right\}dt\\ & =\int \omega \;\left[-\frac{f_{\tau }}{K_{r}}\right]\cdot \exp\left\{j\pi \frac{f_{\tau }^{2}}{K_{r}}\right\}\cdot \exp\left\{-j\frac{4\pi \left(f_{0}+f_{\tau }\right)R(t)}{c}\right\}\\ & \cdot \exp\left\{-j\frac{4\pi vt\sin\varphi }{c}f_{\tau }\right\}\cdot \exp\left\{-j2\pi ft\right\}dt \end{array}$$

By applying the principle of stationary phase (POSP) [26], the 2-D spectrum can be expressed as:

(17) $$S_{3}(f_{\tau },f)=\omega \;\left[-\frac{f_{\tau }}{K_{r}}\right]\cdot \exp\left\{j\pi \frac{f_{\tau }^{2}}{K_{r}}\right\}\cdot \exp\left\{-j4\pi r\left[\cos\varphi \sqrt{p_{f_{\tau }}^{2}-q_{f,f_{\tau }}^{2}}+q_{f,f_{\tau }}\sin\varphi \right]\right\}$$

where:

(18) $$\begin{array}{cc} p_{f_{\tau }}=\frac{f_{0}+f_{\tau }}{c}, & q_{f,f_{\tau }}=\frac{f}{2v}+\frac{f_{\tau }\sin\varphi }{c} \end{array}$$

But *r* is defined in the range time domain, and its range variation cannot be adjusted in the range frequency domain. We need to set the range to its reference one for bulk compensation in the frequency domain. In the traditional Omega-k algorithm, the bulk compensation filter is given in Equation (8). However, according to Equation (17), the term caused by the staggered SAR should be added to the modified bulk compensation filter, which can be expressed as:

(19) $$H_{\text{bulk}}^{\prime }\left(f_{\tau },f\right)=\exp\left\{j4\pi R_{\text{ref}}\left[\cos\varphi \sqrt{p_{f_{\tau }}^{2}-q_{f,f_{\tau }}^{2}}+q_{f,f_{\tau }}\sin\varphi \right]\right\}\cdot \exp\left\{-j\pi \frac{f_{\tau }^{2}}{K_{r}}\right\}$$

After bulk compensation, the residual phase is zero at the reference range, but a residual phase Φ(*f*_τ_, *f*) (including differential RCMC, differential SRC and differential azimuth compression) exists for targets at other ranges [25]:

(20) $$\Phi \left(f_{\tau },f\right)=\exp\left\{j\frac{4\pi \left(r-R_{\text{ref}}\right)}{c}\left[\cos\varphi \sqrt{p_{f_{\tau }}^{2}-q_{f,f_{\tau }}^{2}}+q_{f,f_{\tau }}\sin\varphi \right]\right\}$$

Then, the residual phase is compensated by warping of the range frequency axis, using a modified Stolt interpolation. Combining the residual phase Equation (21), the new substitution range frequency of the modified Stolt interpolation is:

(21) $$f_{0}+f_{\tau }^{*}=\cos\varphi \sqrt{p_{f_{\tau }}^{2}-q_{f,f_{\tau }}^{2}}+q_{f,f_{\tau }}\sin\varphi$$

After the modified Stolt interpolation, the range frequency axis *f*_τ_ is resampled and mapped to a new axis $f_{\tau }^{*}$, and the new 2D spectrum can be expressed as:

(22) $$S_{3\_\text{new}}\left(f_{\tau }^{*},f\right)=\omega \left[-\frac{f_{\tau }}{K_{r}}\right]\cdot \exp\left\{-j\frac{4\pi \left(r-R_{\text{ref}}\right)}{c}\left(f_{0}+f_{\tau }^{*}\right)\right\}$$

As shown in Equation (22), a linear phase is left in the new 2-D spectrum in both directions, which implies that the targets in all range have been focused. Then, an azimuth IFFT operation is performed to transform the new 2D spectrum to the azimuth time and range frequency domain:

(23) $$\begin{array}{ll} S_{4}\left(f_{\tau }^{*},t\right) & =\frac{1}{2\pi }\int_{-B_{a}/2}^{B_{a}/2}S_{3\_\text{new}}\left(f_{\tau }^{*},f\right)\cdot \exp\left\{j2\pi ft_{0}\right\}df\\ & =\frac{1}{2\pi }\omega \left[-\frac{f_{\tau }^{*}}{K_{r}}\right]\cdot \sin c\left(t-t_{0}\right)\cdot \exp\left\{-j\frac{4\pi \left(r-R_{\text{ref}}\right)}{c}\left(f_{0}+f_{\tau }^{*}\right)\right\} \end{array}$$

where *B_a_* and *t_0_* denote the azimuth bandwidth and target azimuth location time, respectively. However, the signal still has geometric distortion after the above processing due to the range-walk effect, so geometric correction is performed in the azimuth time and range frequency domain with the filter $H_{c}(f_{\tau }^{*},t)$ [13]:

(24) $$H_{c}(f_{\tau }^{*},t)=\exp\left\{j2\pi f_{\tau }^{*}\Delta T\left(t\right)\right\}=\exp\left\{j4\pi f_{\tau }^{*}\frac{vt\sin\varphi }{c}\right\}$$

After the above processing, a range IFFT is performed, leading to an accurately focused distortion-free image:

(25) $$\begin{array}{ll} S_{5}\left(\tau ,t\right) & =\frac{1}{4\pi^{2}}\sin c\left(t-t_{0}\right)\int_{-f_{s}^{*}/2}^{f_{s}^{*}/2}S_{4}\left(f_{\tau }^{*},t\right)\cdot H_{c}(f_{\tau }^{*},t)\cdot \exp\left\{j2\pi f_{\tau }^{*}\tau_{0}\right\}df_{\tau }^{*}\\ & =\frac{1}{4\pi^{2}}\sin c\left(t-t_{0}\right)\cdot \mathrm{sinc}\;\left[\tau -\tau_{0}+\frac{2vt\sin\varphi }{c}-\frac{2\left(r-R_{\text{ref}}\right)}{c}\right]\cdot \exp\left\{-j\frac{4\pi \left(r-R_{\text{ref}}\right)}{\lambda }\right\} \end{array}$$

where $f_{s}^{*}$ and **τ**_0_ denote the new range sampling rate and target range location time, and $\tau_{0}=\overset{2R_{\text{ref}}}{\;}/\underset{c}{\;}$. Therefore Equation (25) can be revised as:

(26) $$S_{5}\left(\tau ,t\right)=\frac{1}{4\pi^{2}}\sin c\left(t-t_{0}\right)\cdot \mathrm{sinc}\;\left[\tau +\frac{2\left(vt\sin\varphi -r\right)}{c}\right]\cdot \exp\left\{-j\frac{4\pi \left(r-R_{\text{ref}}\right)}{\lambda }\right\}$$

Finally, an accurately focused distortion-free image is acquired.

## 4. Performance Evaluation

**Figure 7.**
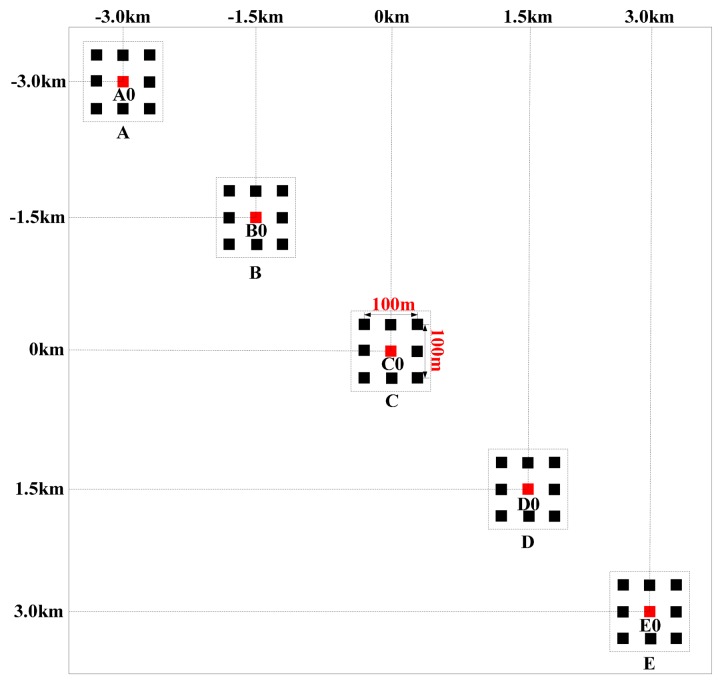
Distribution of the simulation scene.

In this section we first give a computational complexity analysis to our proposed algorithm and then provide some simulation results to verify its performance. The parameters used in our simulations are listed in Table 1 and the scene is shown in Figure 7. All the targets are located in five different regions labeled as A, B, C, D and E, with their centers denoted by A0, B0, C0, D0, and E0, respectively.

### 4.1. Computational Complexity Analysis

The complexity of the traditional and our proposed algorithm in each step is studied in terms of number of floating point operations (FLOP). Each FLOP can either be a real multiplication or a real addition [25]. Assume the sampled echo data has a size of *N_a_* × *N_r_* (azimuth× range), and the Lagrange and Stolt interpolation kernel length are *M_ken_l_* and *M_ken_s_*, respectively. Because of the recovered RCM in the traditional algorithm, the sampled data will go through zero padding processing in the range direction, and we assume the extended range sample number is $N_{r}^{\prime }$. The FLOP in each step is provided in Table 2. Obviously, the storage requirement can be cut down $N_{r}^{\prime }/N_{r}$ times by applying our proposed algorithm.

According to Table 2, the total FLOP of the traditional algorithm is:

(27) $${\text{FLOP}}_{\text{tra}}=N_{a}N_{r}^{\prime }\;\left[20+4M_{\text{ken\_l}}+4M_{\text{ken\_s}}+20\log_{2}N_{r}^{\prime }+10\log_{2}N_{a}\right]$$

While for our proposed algorithm it is:

(28) $${\text{FLOP}}_{\text{pro}}=N_{a}N_{r}\;\left[26+4M_{\text{ken\_l}}+4M_{\text{ken\_s}}+10\log_{2}N_{r}+10\log_{2}N_{a}\right]$$

We can define the computation efficiency factor ζ as:

(29) $$\zeta =\frac{{\text{FLOP}}_{\text{tra}}}{{\text{FLOP}}_{\text{pro}}}$$

Now consider a specific example. Assume the echo data size is 65,536 × 16,384 (*N_a_* × *N_r_*), and the Lagrange interpolation is 3-point (*M_ken_l_* = 3), while the Stolt interpolation is 8-point (*M_ken_s_* = 8). Furthermore, the range sampling number is 65,536 $\left(N_{r}^{\prime }=65,536,N_{r}^{\prime }/N_{r}=4\right)$ in the traditional algorithm. Then the computation efficiency ζ is:

(30) $$\zeta =\frac{N_{r}^{\prime }\;\left[64+20\log_{2}N_{r}^{\prime }+10\log_{2}N_{a}\right]}{N_{r}\;\left[70+10\log_{2}N_{r}+10\log_{2}N_{a}\right]}\approx 6$$

which indicates a significant saving in computational complexity by our proposed algorithm. Even more savings can be achieved in some other situations, such as high-resolution-wide-swath SAR [27].

### 4.2. Simulation Results

**Figure 8.**
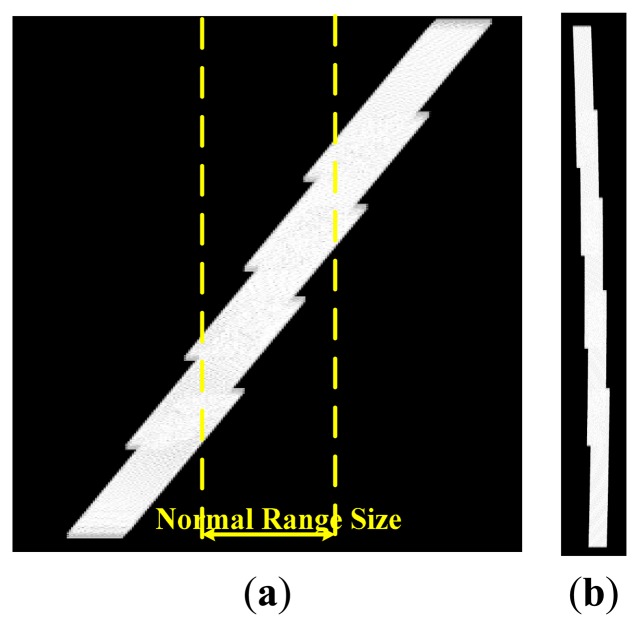
Processing results before the ANI. (**a**) Traditional algorithm; (**b**) Proposed algorithm.

**Figure 9.**
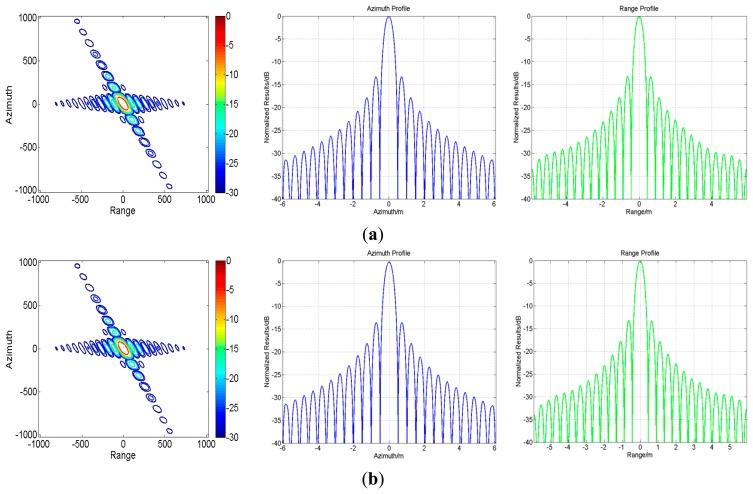
Contour plots and range/azimuth slices for target A0. (**a**) Imaging results of the traditional algorithm; (**b**) Imaging results of our proposed algorithm.

With the parameters listed in Table 1 and the simulation scene shown in Figure 7, the processing results of both the traditional algorithm and our proposed one before ANI are shown in Figure 8. As Figure 8a shows, the range history is recovered back into its normal form in the traditional algorithm. So, zero padding is inevitable in range direction, which would bring pressure on data storage and computation load. However, in Figure 8b, the processing for range-walk recovery is no longer needed and the inherent signal characteristics of staggered SAR are preserved. And at the edge of the scene, there is still a little but tolerable residue range-walk which cannot be removed, as the function of varying PRI is referred to the range-walk of scene center. Then, the imaging results for center A_0_ of A-region are shown in Figure 9. Comparing these two sets of results, we would not be able to see a clear difference between them, indicating that our proposed algorithm has been adequately and equally effective with the traditional one.

**Figure 10.**
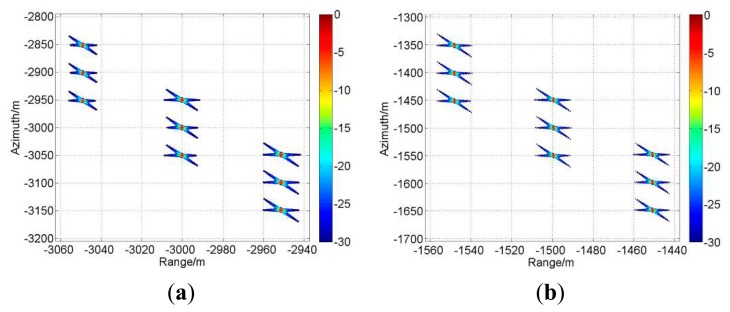

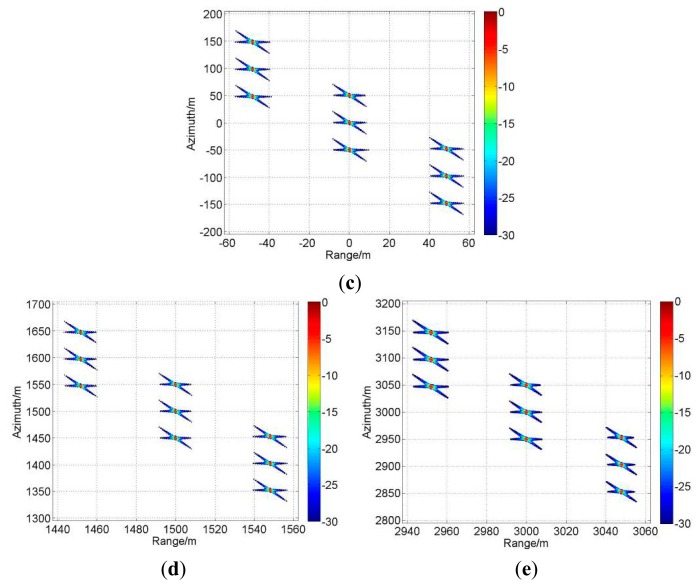
Imaging results for the five different regions. (**a**) Imaging results of A-region; (**b**) Imaging results of B-region; (**c**) Imaging results of C-region; (**d**) Imaging results of D-region; (**e**) Imaging results of E-region.

To show the performance of the proposed algorithm with respect to the spatially variant slant range, the imaging results for all five regions are provided in Figure 10. Moreover, the spatial resolution (azimuth resolution ρ*_a_*, range resolution ρ*_r_*), peak side lobe ratio (PSLR) and integrated side lobe ratio (ISLR) for each simulated region center (A_0_/B_0_/C_0_/D_0_/E_0_) are listed in Table 3. All of these indicate a quality imaging result by our proposed algorithm, which has adapted to the full-scene requirement effectively.

## 5. Conclusions

In this paper, by continuously varying PRI as a function of the linear term of range-walk, the staggered SAR technique was employed for high-speed platform highly-squint SAR applications, and a low-storage low-complexity modified Omega-k algorithm based on ANI was proposed. The formulation of the proposed algorithm was derived based on an approximate model about staggered SAR and sliding receive-window SAR, with only a residual phase error left due to the effect of a time-varying Doppler phase. And this residual phase error can be easily compensated at the beginning of the imaging process. Then, considering the large *fd_0_* in high-speed platform highly-squint staggered SAR, optimal ANI processing based on baseband operation is adopted to resample the data back to a uniform grid. Unlike the direct interpolation operation, in this approach the center of azimuth spectrum is moved to zero-frequency first to guarantee the validity and effectiveness of Lagrange interpolation. Subsequently, novel bulk compression and Stolt interpolation was proposed, considering the signal feature changed by staggered SAR. As shown by our analysis and simulation results, the computational complexity of the proposed algorithm is significantly lower than the traditional one, without any observable loss of performance in terms of imaging quality.

## Figures and Tables

**Table 1.** Main simulation parameters.

**Parameters**	**Value**	**Parameters**	**Value**	**Parameters**	**Value**
**λ (cm)**	1.875	***v* (m/s)**	350	***f_s_* (MHz)**	180
***f_0_* (GHz)**	16.0	**φ (°)**	65.0	**height of platform (Km)**	8.0
***PRI_0_* (ms)**	0.5	***R_ref_* (Km)**	143.9	***Range Chirp FM rate* (s^−2^)**	7.5*e^13^*
***Look Angle* (°)**	86.8	***fd_0_* (Hz)**	−33,854.9	***Antenna Length* (m)**	0.8

**Table 2.** Comparison of computational complexity [25].

	**Traditional Algorithm**	**Proposed Algorithm**
**Range FFT**	$5N_{a}N_{r}^{\prime }\log_{2}N_{r}^{\prime }$	0
**RCM Recovery (Residual phase compensation)**	$6N_{a}N_{r}^{\prime }$	6*N_a_N_r_*
**Range IFFT**	$5N_{a}N_{r}^{\prime }\log_{2}N_{r}^{\prime }$	0
**Baseband-Shift**	$6N_{a}N_{r}^{\prime }$	6*N_a_N_r_*
**Lagrange interpolation**	$2\left(2M_{\text{ken\_l}}-1\right)N_{a}N_{r}^{\prime }$	2(2*M_ken_l_*−1)*N_a_N_r_*
**Band Recovery**	$6N_{a}N_{r}^{\prime }$	6*N_a_N_r_*
**Range FFT**	$5N_{a}N_{r}^{\prime }\log_{2}N_{r}^{\prime }$	5*N_a_N_r_*log_2_*N_r_*
**Azimuth FFT**	$5N_{a}N_{r}^{\prime }\log_{2}N_{a}$	5*N_a_N_r_*log_2_*N_a_*
**Bulk compression**	$6N_{a}N_{r}^{\prime }$	6*N_a_N_r_*
**Stolt interpolation**	$2\left(2M_{\text{ken\_s}}-1\right)N_{a}N_{r}^{\prime }$	2(2*M_ken_s_*−1)*N_a_N_r_*
**Azimuth IFFT**	$5N_{a}N_{r}^{\prime }\log_{2}N_{a}$	5*N_a_N_r_*log_2_*N_a_*
**Geometric correction**	0	6*N_a_N_r_*
**Range IFFT**	$5N_{a}N_{r}^{\prime }\log_{2}N_{r}^{\prime }$	5*N_a_N_r_*log_2_*N_r_*

**Table 3.** Imaging quality analysis for the five point targets.

	**Azimuth** *			**Range** *		
	
	**ρ*_a_* (m)**	**PSLR (dB)**	**ISLR (dB)**	**ρ*_r_* (m)**	**PSLR (dB)**	**ISLR (dB)**
**A_0_**	0.962	−13.11	−10.01	0.886	−13.21	−10.05
**B_0_**	0.951	−13.19	−10.11	0.886	−13.22	−10.06
**C_0_**	0.946	−13.26	−10.10	0.886	−13.25	−10.10
**D_0_**	0.950	−13.20	−10.12	0.887	−13.23	−10.05
**E_0_**	0.959	−13.12	−10.03	0.887	−13.22	−10.04

* Ideal azimuth resolution is 0.946 m, ideal range resolution is 0.886 m.
